# Longitudinal Change of Choroidal Thickness after Pars Plana Vitrectomy for Idiopathic Epiretinal Membrane

**DOI:** 10.3390/jcm11236950

**Published:** 2022-11-25

**Authors:** Dong-Ik Kim, Ki-Woong Bae, Daniel Duck-Jin Hwang

**Affiliations:** 1 Department of Ophthalmology, HanGil Eye Hospital, Incheon 21388, Republic of Korea; 2 Department of Ophthalmology, Catholic Kwandong University College of Medicine, Incheon 22711, Republic of Korea

**Keywords:** choroidal thickness, pars plana vitrectomy, air tamponade, idiopathic epiretinal membrane

## Abstract

This study aimed to investigate changes in choroidal thickness after pars plana vitrectomy (PPV) with and without air tamponade in patients with idiopathic epiretinal membrane (ERM). We retrospectively reviewed 61 patients with ERM who underwent a 25-gauge transconjunctival sutureless PPV. The patients were divided into two groups: the air tamponade group (30 eyes) and the nontamponade group (31 eyes). Subfoveal choroidal thickness (SFCT) was analyzed over 12 months. No significant differences were seen between the two groups at baseline. For all patients, the SFCT was significantly thicker at 1 month after surgery and significantly thinner at 6 and 12 months after surgery than preoperative values. In the subgroup analysis, there was no significant difference in SFCT 3 months after surgery compared with the preoperative values in both groups, but SFCT was significantly lower 6 months after surgery in both groups. In conclusion, our results showed that choroidal thickness temporarily increased after surgery and then gradually decreased until 12 months after the PPV for ERM.

## 1. Introduction

The epiretinal membrane (ERM) is characterized by fibrocellular proliferation on the inner surface of the retina. It is a common macular disorder, and its prevalence increases with age. It causes retinal folds, traction, and macular edema, which result in vision reduction and metamorphopsia [1,2]. Most ERMs are idiopathic but may also occur secondary to retinal vascular disease, retinal break, retinal detachment, intraocular inflammation, or previous intraocular surgery [3].

Surgery involves pars plana vitrectomy (PPV) with membrane peeling. It is known to improve the central retinal thickness and visual acuity when retinal traction and ERM-causing edema resolve after surgery [4,5,6]. However, information remains limited about long-term changes in choroidal thickness after vitrectomy. Some studies have shown that the choroid thickness decreases after surgery [7,8,9,10], whereas other studies have shown there is no change until 12 months after surgery [11].

In this study, we analyzed the postoperative changes in choroidal thickness in patients who underwent vitrectomy for ERM. Subgroup analysis was performed by dividing the cases into air tamponade and nontamponade groups to determine the difference in choroidal thickness depending on whether air tamponade was performed. Furthermore, when optical coherence tomography (OCT) examination of the fellow eyes was performed, the changes in choroidal thickness over time were compared between the operated and fellow eyes.

## 2. Materials and Method

### 2.1. Subjects

This was a retrospective study. The medical records of patients who underwent PPV for idiopathic ERM treatment at Hangil Eye Hospital from October 2015 to February 2020 were reviewed. ERM was clinically diagnosed by fundus examination and confirmed using spectral domain OCT (SD-OCT, Spectralis Version 5.6.1.0, Heidelberg Engineering, Heidelberg, Germany). Patients who were followed up for >12 months after surgery were included. Patients with other diseases that might affect the subfoveal choroidal thickness (SFCT) and macular thickness, such as central serous chorioretinopathy, diabetic retinopathy, retinal vein occlusion, and age-related macular degeneration, were excluded.

### 2.2. Surgical Methods

All the surgeries were performed by a single surgeon (DDH). A standard 3-port 25-gauge vitrectomy was performed using the Constellation Vision Surgical System (Alcon Surgical, Fort Worth, TX, USA). Cataract surgery was combined with vitrectomy in all phakic patients. After core and peripheral vitrectomy, the removal of the ERM and the peeling of the internal limiting membrane were carefully performed using retinal forceps. In some cases, preservative-free triamcinolone (MaQaid^®^; Wakamoto Pharmaceutical Co., Ltd., Tokyo, Japan) was used for membrane removal. After membrane removal, a 360-degree endolaser was routinely performed. In cases of atrophic holes or lattice degeneration, a full air tamponade was performed. Patients who underwent air tamponade were recommended to remain in the prone position for 1 postoperative day. The surgery was completed after confirming there were no remaining membranes or macular holes using intraoperative OCT (RESCAN 700, Zeiss, Germany).

### 2.3. Ophthalmic Examinations

Ophthalmologic examinations, including slit lamp examination, fundoscopy, best-corrected visual acuity (BCVA), central macular thickness (CMT), and SFCT, were performed at the baseline visit, 1 month, 3 months, 6 months, and 1 year after surgery. The CMT was obtained from the central 1 mm subfield in the macular thickness map. The SFCT was measured using the built-in program’s caliper function at a point just below the fovea on a horizontal cross-sectional image of the macula. The vertical distance from the outer border of the hyperreflective line, which corresponds to the retinal pigment epithelium, to the choroidal scleral interface was measured by two independent observers (DIK and KWB), and the average value of the measured thickness was used for analysis.

### 2.4. Ethics Statement

This retrospective study was approved by the Institutional Review Board of Hangil Eye Hospital (IRB No. 22002). All study procedures adhered to the principles of the Declaration of Helsinki. The need for written informed consent was waived because the study involved no more than minimal risk to the subjects.

### 2.5. Statistical Analysis

All statistical analyses were performed using SPSS version 25.0 (IBM Corp., Armonk, NY, USA). Patients were divided into two groups on the basis of whether they had received air tamponade: air tamponade group and nontamponade group. The characteristics of the two groups were compared using independent t-tests and Pearson’s chi-squared tests. A paired t-test was used to compare the values before and after surgery. Statistical significance was established at *p* < 0.05.

## 3. Results

The study included 61 eyes, including 30 and 31 eyes in the air tamponade and nontamponade groups, respectively. There were no significant differences in age, sex, BCVA, CMT, or SFCT between the two groups at baseline (Table 1). There were no cases of postoperative hypotony, vitreous hemorrhage, or endophthalmitis in either group. None of the patients required reoperation owing to complications, such as retinal detachment or ERM recurrence.

### 3.1. Temporal Changes in BCVA and CMT after the Removal of ERM with and without Air Tamponade

BCVA and CMT were significantly different before and after surgery. BCVA improved from 0.29 ± 0.19 logarithm of the minimum angle of resolution (logMAR) to 0.16 ± 0.13 logMAR in the air group (*p* < 0.001), and from 0.26 ± 0.20 logMAR to 0.17 ± 0.15 logMAR in the nontamponade group (*p* = 0.010) (Table 2). CMT decreased from 401.6 ± 75.8 μm to 361.0 ± 43.4 μm in the air group (*p* < 0.001), and from 373.3 ± 62.8 μm to 355.7 ± 41.5 μm in the nontamponade group (*p* = 0.032) (Table 2). There was no significant difference in BCVA, CMT, and SFCT between the two groups 1 year after surgery.

### 3.2. Temporal Changes in SFCT after Removal of ERM with and without Air Tamponade

**Figure 1:**
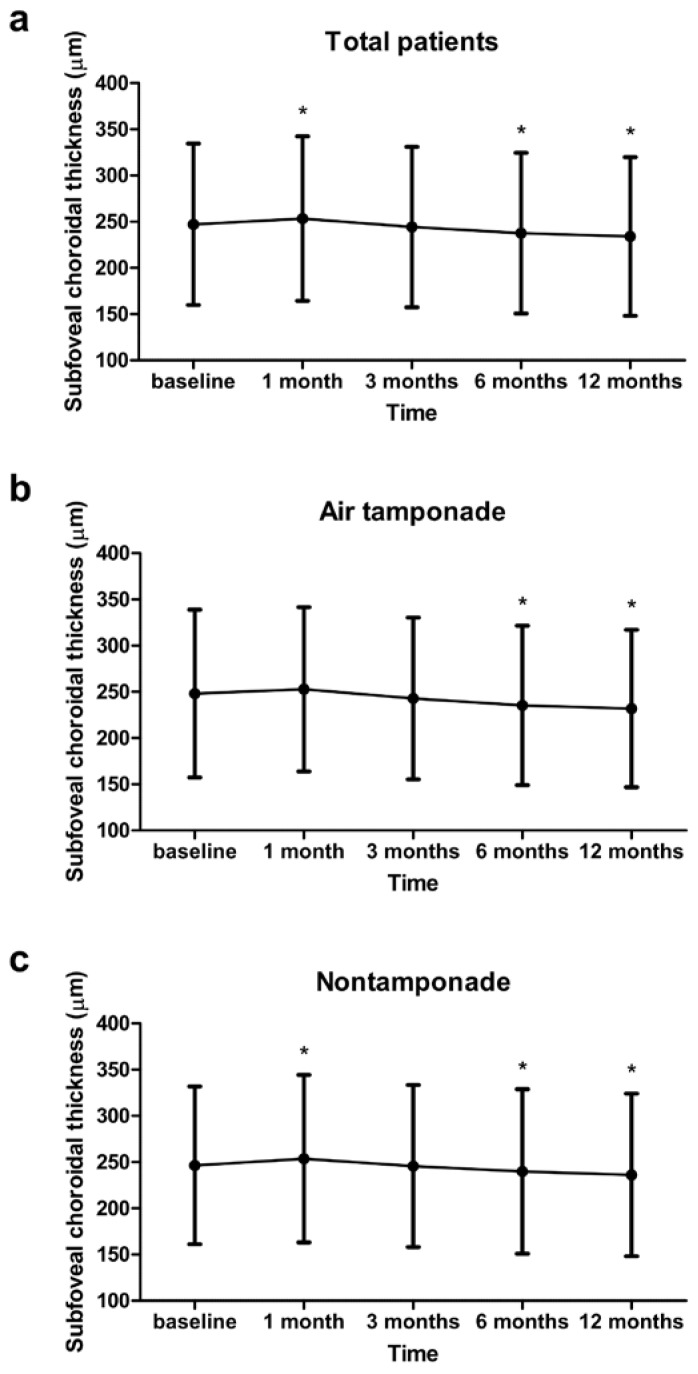
Changes in subfoveal choroidal thickness over 1 year. (**a**) Total patients, (**b**) air tamponade group, and (**c**) nontamponade group. There was no significant difference between the two groups at 1 year. In both groups, the subfoveal choroidal thickness significantly decreased at 6 months and 1 year after the operation compared with the preoperative values. * *p* < 0.05 by paired *t*-test.

For all patients, the SFCT was significantly thicker 1 month after surgery (253.3 ± 89.0 μm, *p* = 0.012) and significantly thinner 6 (237.6 ± 86.9 μm, *p* = 0.003) and 12 months (234.0 ± 85.9 μm, *p* < 0.001) after surgery than the preoperative SFCT (247.2 ± 87.4 μm). However, compared with the preoperative SFCT values, there were no significant difference in those 3 months (244.3 ± 86.9 μm, *p* = 0.240) after surgery. In the air tamponade group, there was no significant difference between SFCT before surgery and that 1 (*p* = 0.184) and 3 months (*p* = 0.236) after surgery, but it decreased significantly 6 (*p* = 0.033) and 12 months (*p* = 0.006) after surgery. The SFCT of the nontamponade group significantly increased 1 month (*p* = 0.028) after surgery and significantly decreased 6 (*p* = 0.024) and 12 months (*p* < 0.001) after surgery. However, there was no significant difference at 3 months (*p* = 0.781) after surgery (Figure 1).

### 3.3. Comparison of SFCT between the Study Eye and the Fellow Eye

Twenty-nine patients in the air group and 25 in the nontamponade group had OCT results in their fellow eye. In these patients, we compared the SFCT between the study and fellow eyes (Table 3). For all patients, there were no differences between the two eyes before surgery; however, 6 and 12 months after surgery, the SFCT of the study eye was significantly thinner than that of the fellow eye (*p* = 0.002 and 0.001, respectively). The nontamponade group showed similar results (*p* = 0.017 and 0.011, respectively). In the air group, the SFCT of the study eye was thinner than that of the fellow eye 6 and 12 months after surgery, but it was only statistically significant 12 months after surgery (*p* = 0.059 and 0.028, respectively).

## 4. Discussion

In this study, we investigated the changes in SFCT over time in patients who underwent PPV surgery with ERM. One month after surgery, SFCT increased significantly, and then started to gradually decrease. Six months after surgery, SFCT decreased significantly compared with that before surgery. SFCT decreased significantly 6 and 12 months after surgery in the group that received air tamponade during surgery and the group that did not, respectively.

These results are similar to those of previous studies. In a prospective study of 21 patients with idiopathic ERM [7], the thickness of the choroid was significantly reduced 3 months after surgery compared with that before surgery. Another prospective study of 53 eyes reported that choroidal thickness decreased significantly in the subfoveal region and in the nasal and temporal quadrants 3 months after surgery [8]. In the case of ERM with vitreomacular traction [9] and ERM with membrane contraction [10], choroidal thickness was significantly reduced 6 months after surgery compared with that before surgery. Meanwhile, in a retrospective study [11] that observed 95 eyes up to 1 year after surgery, choroidal thickness was significantly increased 1 week after vitrectomy compared to that of the baseline value, but there was no difference between the thickness 1 month and 12 months after surgery. Another study of 64 patients also reported that there were no significant differences in choroidal thickness values between preoperative and those up to 3 months after the operation [12]. The difference between these studies is thought to be due to the difference in the degree of ERM before surgery, surgical method, and postoperative follow-up period. A previous study reported a temporary increase in choroidal thickness after surgery [11], but all studies reported that choroidal thickness decreased over time after surgery or there was no difference from that before surgery [7,8,9,10,11,12]. The present study also showed a temporary increase in choroid thickness 1 month after surgery and a decrease at 6 and 12 months after surgery compared with preoperative values. Thus, our study showed results that are similar to those of previous studies.

Animal experiments have shown that proinflammatory prostaglandins and cytokines in the retina increase as an inflammatory response after cataract surgery [13], and previous studies also reported that choroidal thickness increases after cataract surgery [14,15]. Choroidal thickness was thought to temporarily increase 1 month after surgery via this mechanism. Later, owing to the removal of the vitreous body and ERM, the oxygen and nutrient supply to the retinal tissues improve, and the thickness of the choroid is expected to decrease. Taking into account studies that showed no change in choroidal thickness before and after vitrectomy in patients with macular hole [11,12,16] and patients with rhegmatogenous retinal detachment [17,18], the effect of internal limiting membrane peeling and vitreous removal on choroidal thickness is not considered significant. Therefore, the decrease in choroidal thickness after surgery in this study was considered an effect of ERM removal. Additionally, given that there was no difference in SFCT due to the presence or absence of air tamponade during surgery, it can be considered that postoperative SFCT was not affected by air tamponade.

In our study, there was no difference in SFCT between the healthy fellow eye and the study eye before surgery. Previous studies have not reported any difference in choroidal thickness between eyes before surgery [7,8]. The authors stated that ERM was not related to choroidal thickness and vice versa [8]. A recent study found no difference in choroidal thickness between the eyes of patients with ERM without membrane contraction, but the macular choroidal thickness in patients with ERM with membrane contraction was significantly higher than that of their fellow eyes [10]. Macular traction caused by ERM would increase the dilatation and tortuosity of retinal blood vessels, causing abnormalities in the supply of oxygen and nutrients to the retina. These changes cause secondary choroidal vasodilation, which causes the thickening of the choroid. Studies using fluorescence angiography have confirmed that retinal capillary blood flow velocity is reduced in eyes with ERM [19,20]. However, of the 84 eyes included in that study, only 22 required surgeries. Therefore, there are limitations in interpreting the results. In each of the reported studies [7,8,10], the preoperative status of patients with ERM were different, and further studies with more patients are needed because only a small number of patients were analyzed.

This study has several limitations, such as its retrospective design and small sample size. No qualitative analysis of ERM was performed before surgery, and there was a selection bias because only patients who were followed up for more than 12 months after surgery were included. There is diurnal variation in choroidal thickness [21,22]; however, it is impossible to control the diurnal variation. Laser-induced inflammation may have affected choroidal thickness. However, since the 360-degree endolaser was applied to all patients, the effect of the laser on the subgroup analysis is considered to be insignificant. Lastly, the thickness of the choroid was measured at one point, namely, the subfovea.

The strength of our study was that all surgeries were performed by an experienced retinal specialist, and changes in SFCT were observed over a relatively long period of 12 months. Furthermore, although randomization was not performed, the effect of air tamponade on the postoperative choroidal thickness was analyzed.

In conclusion, our results showed that the thickness of the choroid temporarily increased after surgery and then gradually decreased in the eyes treated with PPV for ERM. After vitrectomy and membrane peeling, exposure of the ocular tissue to a higher oxygen concentration environment may constrict the choroidal vessels and reduce the thickness of the choroid. The same results were observed in the with and without air tamponade groups.

## Figures and Tables

**Table 1:** Comparison of patient characteristics at baseline.

Parameter	Total Patients (*n* = 61)	Air Tamponade (*n* = 30)	Nontamponade (*n* = 31)	*p*-Value
Age	63.6 ± 9.3	63.6 ± 8.5	63.5 ± 10.1	0.961 *
Sex (male/female)	21/40	10/20	11/20	0.860 ^†^
Operated eye (right/left)	32/29	14/16	18/13	0.373 ^†^
BCVA (logMAR)	0.27 ± 0.19	0.29 ± 0.19	0.26 ± 0.20	0.573 *
CMT (μm)	387.2 ± 70.4	401.6 ± 75.8	373.3 ± 62.8	0.117 *
SFCT (μm)	247.2 ± 87.4	248.1 ± 90.8	246.4 ± 85.4	0.941 *

Values are presented as n or mean ± standard deviation. BCVA, best-corrected visual acuity; logMAR, logarithm of the minimum angle of resolution; CMT, central macular thickness; SFCT, subfoveal choroidal thickness. * *p*-values were derived using independent *t*-tests. ^†^ *p*-values were derived using Pearson’s chi-squared tests.

**Table 2:** Comparison of the parameters of the air tamponade and nontamponade groups over 1 year.

Parameter	Group	Baseline	1 Month	3 Months	6 Months	1 Year	*p*-Value ^†^
**BCVA (logMAR)**	Air tamponade	0.29 ± 0.19	0.29 ± 0.19	0.21 ± 0.12	0.17 ± 0.10	0.16 ± 0.13	<0.001
	Nontamponade	0.26 ± 0.20	0.28 ± 0.22	0.21 ± 0.21	0.20 ± 0.18	0.17 ± 0.15	0.010
	*p*-value *	0.573	0.896	0.934	0.417	0.923	
**CMT (** **μm)**	Air tamponade	401.6 ± 75.8	389.4 ± 57.7	381.6 ± 49.8	369.1 ± 43.6	361.0 ± 43.4	<0.001
	Nontamponade	373.3 ± 62.8	381.0 ± 59.0	378.5 ± 44.3	368.2 ± 44.5	355.7 ± 41.5	0.032
	*p*-value *	0.117	0.575	0.797	0.939	0.626	
**SFCT (** **μm)**	Air tamponade	248.1 ± 90.8	252.8 ± 88.9	242.9 ± 87.5	235.4 ± 86.2	231.9 ± 85.1	0.006
	Nontamponade	246.4 ± 85.4	253.7 ± 90.6	245.7 ± 87.7	239.8 ± 89.0	236.1 ± 87.9	<0.001
	*p*-value *	0.941	0.967	0.901	0.844	0.848	

Values are presented as mean ± standard deviation. BCVA, best-corrected visual acuity; logMAR, logarithm of the minimum angle of resolution; CMT, central macular thickness; SFCT, subfoveal choroidal thickness. * *p*-values were derived using independent *t*-tests. ^†^ *p*-values were derived using paired *t*-tests for the preoperative and postoperative 1 year values.

**Table 3:** Comparison of the thickness of the subfoveal choroidal between the study and fellow eyes in the air tamponade and nontamponade groups over 1 year.

Group		Baseline	6 Months	1 Year
**Total patients** **(*n* = 54)**	Study eye	250.4 ± 90.2	239.2 ± 90.1	235.8 ± 89.0
	Fellow eye	261.5 ± 89.5	261.0 ± 89.3	260.6 ± 89.5
	*p*-value *	0.123	0.002	0.001
**Air tamponade** **(*n* = 29)**	Study eye	249.9 ± 91.8	237.0 ± 87.2	233.4 ± 86.2
	Fellow eye	254.8 ± 92.4	254.3 ± 92.0	254.3 ± 92.1
	*p*-value *	0.611	0.059	0.028
**Nontamponade** **(*n* = 25)**	Study eye	250.9 ± 90.2	241.8 ± 95.1	238.5 ± 93.8
	Fellow eye	269.3 ± 87.3	268.6 ± 87.4	267.8 ± 87.7
	*p*-value *	0.099	0.017	0.011

Values are presented as mean ± standard deviation. * *p*-values were derived using paired *t*-tests.

## Data Availability

The data are not available for public access because of patient privacy concerns but are available from the corresponding author upon reasonable request.
